# When Sex Matters: Differences in the Central Nervous System as Imaged by OCT through the Retina

**DOI:** 10.3390/jimaging10010006

**Published:** 2023-12-25

**Authors:** Ana Nunes, Pedro Serranho, Pedro Guimarães, João Ferreira, Miguel Castelo-Branco, Rui Bernardes

**Affiliations:** 1 Coimbra Institute for Biomedical Imaging and Translational Research (CIBIT), Institute of Nuclear Sciences Applied to Health (ICNAS), University of Coimbra, 3000-548 Coimbra, Portugal; anunes@fmed.uc.pt (A.N.);; 2 Department of Sciences and Technology, Universidade Aberta, 1269-001 Lisboa, Portugal; 3 Faculty of Sciences and Technology, University of Coimbra, 3030-201 Coimbra, Portugal; 4 Clinical Academic Center of Coimbra (CACC), Faculty of Medicine, University of Coimbra, 3000-548 Coimbra, Portugal

**Keywords:** texture analysis, neuroretina, central nervous system, sex differences, interocular asymmetry, optical coherence tomography

## Abstract

Background: Retinal texture has gained momentum as a source of biomarkers of neurodegeneration, as it is sensitive to subtle differences in the central nervous system from texture analysis of the neuroretina. Sex differences in the retina structure, as detected by layer thickness measurements from optical coherence tomography (OCT) data, have been discussed in the literature. However, the effect of sex on retinal interocular differences in healthy adults has been overlooked and remains largely unreported. Methods: We computed mean value fundus images for the neuroretina layers as imaged by OCT of healthy individuals. Texture metrics were obtained from these images to assess whether women and men have the same retina texture characteristics in both eyes. Texture features were tested for group mean differences between the right and left eye. Results: Corrected texture differences exist only in the female group. Conclusions: This work illustrates that the differences between the right and left eyes manifest differently in females and males. This further supports the need for tight control and minute analysis in studies where interocular asymmetry may be used as a disease biomarker, and the potential of texture analysis applied to OCT imaging to spot differences in the retina.

## 1. Introduction

In light of the thoroughly researched relationship between the eye and the brain [1,2,3,4], sex differences in brain size have been hypothesised [5] to be associated with reports of sexual dimorphism in visual perception. Interestingly, lateral hemisphere activation during visual perception tasks was found to be independent of sex, handedness, and ocular dominance [6], the latter being associated with interocular retinal thickness asymmetries [7].

The difference between the right and left eyes of the same individual is a phenomenon of interest where retinal biomarkers are concerned. The difference between the two eyes is a key factor to consider when interpreting retinal thickness values in ocular disorders such as glaucoma [8,9], particularly in its early stages, when both eyes still yield measurements within normative values. 

When it comes to the healthy population, a certain degree of retinal thickness symmetry is often assumed. While some studies confirm this symmetry assumption [8,10], others report statistically significant differences between healthy individuals’ right and left eyes [9,11]. Notably, Jacobsen et al. [12] found a slight age and sex effect on retinal thickness interocular differences but advised caution in considering the magnitude of such an effect. Furthermore, a few studies in pediatric subjects have reported no sex effects on interocular thickness symmetry [13,14,15]. Overall, sex remains largely overlooked when comparing right and left eyes in healthy adults. 

The literature on sex differences found in retinal structure is focused mostly around single-layer or full retina thickness, as measured by optical coherence tomography (OCT) [10,16,17,18,19]. OCT is a non-invasive imaging technique used for the visualization of the microstructure of various biological tissues in vivo and in situ. In clinical settings, OCT imaging plays an important role in the diagnosis of ocular disorders. Furthermore, this medical imaging technique is increasingly employed in neuroimaging research, where the potential of using the retina as a window to the brain [1,2,3,4] for the diagnosis of neurodegenerative disorders such as Alzheimer’s disease [20,21], Parkinson’s disease [22,23], and multiple sclerosis [24,25], is being explored.

Several OCT-based studies have reported differences in retinal thickness between women and men. Men are often reported to have thicker retinas than women [10,16,17], although some conflicting results have been found [8,17]. In their study, Ooto and Yoshimura [18] hypothesised that the increased retinal thickness in men might be associated with a relatively larger eye size. On the other hand, Delori et al. [19] discusses how sex differences in the shape and size of the fovea may affect thickness measurements. However, non-anatomical factors may play an even larger role in the sexual dimorphism of the human retina. The relationship between various retinopathies and gonadal hormones has been addressed, as in [26,27], where the authors highlighted the role of sex hormones on the pathophysiology of ocular disorders, such as age-related macular degeneration and diabetic retinopathy. Despite the possible connection between gonadal hormones and the health/disease status of the retina, the intricate interplay between sex and vision both in healthy individuals and across different disease processes [5] suggests that observed sex differences in the retina cannot be attributed to a single mechanism [28]. 

Ultimately, studying the differences between the right and left eyes in the healthy population helps determine the normal demographic variations, allowing pathological deviations to be correctly identified. Indeed, for some disorders, interocular asymmetries have been used as a biomarker of disease [29,30]. Although thickness measurements are usually the metric of choice, interocular differences have also been identified in non-thickness measurements, such as vessel diameter [11]. Another example is the topography of the foveal centre mosaic, where a slight sex asymmetry was found [31]. 

In recent years, the use of yet another type of information–image texture–from OCT data has been introduced [32,33]. Texture plays an important role in identifying objects and understanding complex scenes [34,35]. The concept of image or visual texture is challenging to define, and different definitions have been proposed in the literature. A generally agreed-upon description for image texture defines it as the spatial arrangement of an image’s pixels’ grey-level/intensity/brightness values or colour [36,37,38]. 

Texture analysis is thus an umbrella term encompassing different approaches aimed at quantifying or describing the spatial distribution of grey levels or colour within an image. The field of texture analysis has been gaining momentum as a source of valuable biomarkers for neurodegeneration. The use of texture-based methods as a tool to assess the central nervous system (CNS) status from medical neuroimaging data is based on the premise that medical images contain a quantifiable texture “signature” [37] that is specific to a particular biological process or state, be it healthy (normal) or pathological. Where retinal OCT imaging is concerned, texture analysis methods have been employed to explore structural alterations resulting from specific ophthalmological (e.g., glaucoma [32]) and neurodegenerative disorders (e.g., Alzheimer’s disease and Parkinson’s disease [33], and multiple sclerosis [24,25]), and to characterise the healthy (normal) ageing process [39]. 

Out of the wide variety of texture-based methods available, the grey-level co-occurrence matrix (GLCM) method stands out for its popularity and broad applicability. This method was first proposed by Haralick et al. [36] in 1973. Despite its early development, it remains, to this day, a benchmark method in comparative studies that employ texture ensemble approaches. 

The GLCM texture analysis method starts by computing one or more co-occurrence matrices from the images under study. In a co-occurrence matrix, pairs of grey-level values of image pixels with a specific spatial relationship (e.g., two adjacent pixels in the horizontal direction) are tabulated. Typically, four different matrices are computed for the same inter-pixel distance, corresponding to four different pixel pair orientations: 0°, 45°, 90°, and 135°. Texture features are then computed from these matrices to characterise the local grey-level variations within the image.

We propose using texture metrics derived from the GLCM to assess the sex effect on interocular differences in the healthy population, as texture-based methods can detect discrepancies in the retina that are not conveyed by thickness measurements [24,25,32,33]. 

In this study, we investigate the differences in texture of the neuroretina between healthy individuals’ right and left eyes. More specifically, we demonstrate how interocular differences manifest differently in men and women, as quantified by retinal GLCM-based texture features. Furthermore, focusing specifically on the six layers of the neuroretina, these variations highlight sex differences in the CNS. 

## 2. Materials and Methods

All image processing and statistical analysis were performed using Matlab R2022a (The MathWorks Inc., Natick, MA, USA) running on a Ubuntu operating system desktop computer.

### 2.1. Data Collection

The data were gathered from the authors’ institutional database. The studies from which data were collected were approved by the Faculty of Medicine of the University of Coimbra Ethics Committee, performed according to the tenets laid out in the Declaration of Helsinki [40], and informed consents were obtained from all participants. All data herein were anonymized. 

Both eyes of 98 age-matched and sex-balanced healthy controls with no known retinal pathology were imaged by Cirrus HD-OCT 5000 (Carl Zeiss Meditec, Dublin, CA, USA) scanner, using the 512 × 128 macular cube protocol centred on the macula. The same operator performed all acquisitions. All participants had normal visual acuity (≥0.8 in each eye), a sphere between ±5 diopters, and a cylinder between ±3 diopters. None of the participants had a medical history of ophthalmological, neurological, or other systemic diseases. For more details on group demographics, please refer to Table 1. 

### 2.2. Image Processing

The OCT Explorer software (Retinal Image Analysis Lab, Iowa Institute for Biomedical Imaging, Iowa City, IA, USA) [41,42,43] was used to segment the six layers of the neuroretina: the retinal nerve fibre layer (RNFL), the ganglion cell layer (GCL), the inner plexiform layer (IPL), the inner nuclear layer (INL), the outer plexiform layer (OPL), and the outer nuclear layer (ONL).

**Figure 1:**
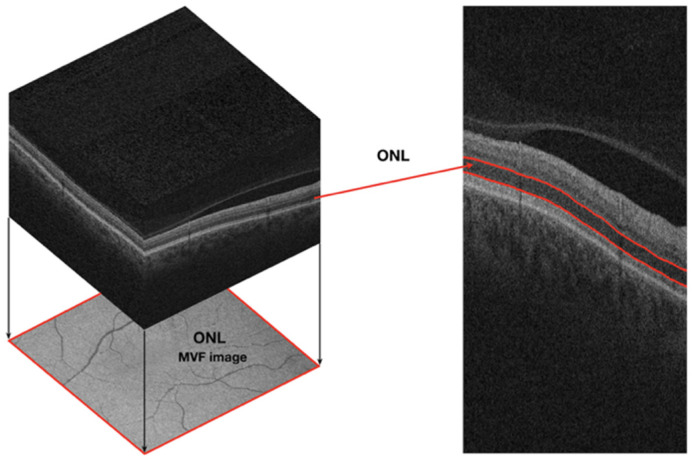
Representation of a mean value fundus image (MVF) computed for the outer nuclear layer (ONL) of the right eye of a 54-year-old female participant regarding the optical coherence tomography volumetric data acquired. These images are shown for reference purposes only; the MVF image projection was intensity-corrected for ease of visualisation.

**Figure 2:**
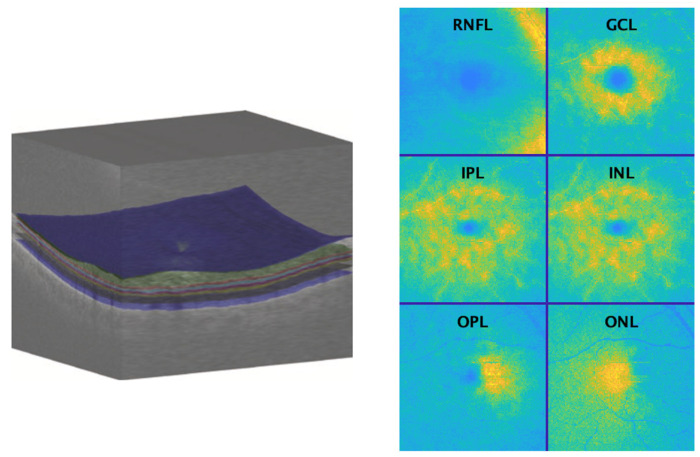
An optical coherence tomography volume (**left**) is shown with interfaces displayed in colour, e.g., the interface vitreous-retina in blue. Pseudo-colour fundus images are (**right**) shown for retinal layers of the right eye of a 54-year-old female participant: the retinal nerve fibre layer (RNFL), the ganglion cell layer (GCL), the inner plexiform layer (IPL), the inner nuclear layer (INL), the outer plexiform layer (OPL), and the outer nuclear layer (ONL). Pseudo-colour is used for the ease of visualisation only.

A 2D mean value fundus (MVF) reference image for each of the six neuroretina layers was computed from the 3D OCT data of each eye scan, following the approach in [44]. In this image, each pixel is the depth-wise average of the A-scan values located between the two retinal layer interfaces that delineate the layer of interest (see Figure 1 and Figure 2). 

**Figure 3:**
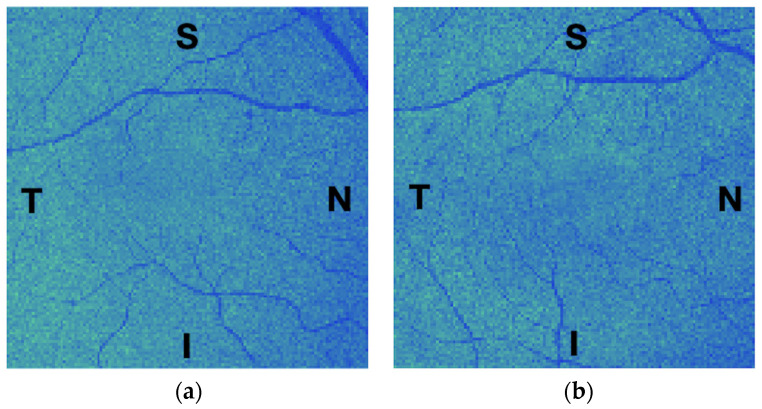
Example of the mean value fundus (MVF) images computed for the outer nuclear layer of a 54-year-old female participant: (**a**) the right eye; and (**b**) the left eye, flipped to match the regions of the right eye. S, T, N, and I stand for the eye’s superior, temporal, nasal, and inferior regions, respectively. These images are shown for reference purposes only; they were intensity-corrected and pseudo-colour-coded for ease of visualisation.

MVF images of left eyes were flipped horizontally so that the temporal and nasal regions were consistent across all eye scans. This ensures that comparative analysis of the computed texture features considers metrics originating from the same relative position across eyes (see Figure 3).

### 2.3. Texture Analysis

In this work, GLCM-based texture features were computed for each of six neuroretina MVF images, following the previously used approach of [33,39]. The computation of texture metrics from fundus reference images is a unique approach to OCT data analysis–one that is significantly distinct from the traditional approach of measuring the thickness of the retina (or that of specific retinal layers). A prior study [33] showed that texture-based metrics computed from retinal MVF images are not simply a surrogate for OCT thickness measurements. It was also discussed how these texture-based features could arguably be more discriminative than retinal thickness measurements, as they are able to capture multivariate information; thickness-based measurements, on the other hand, only allow for univariate comparisons.

The MVF images, originally of 512 × 128 pixels, were first down-sampled to 128 × 128 to obtain isotropic sampling in both the horizontal and vertical directions. These images were also converted to 16 grey levels to reduce the size of the GLCM matrices. 

**Figure 4:**
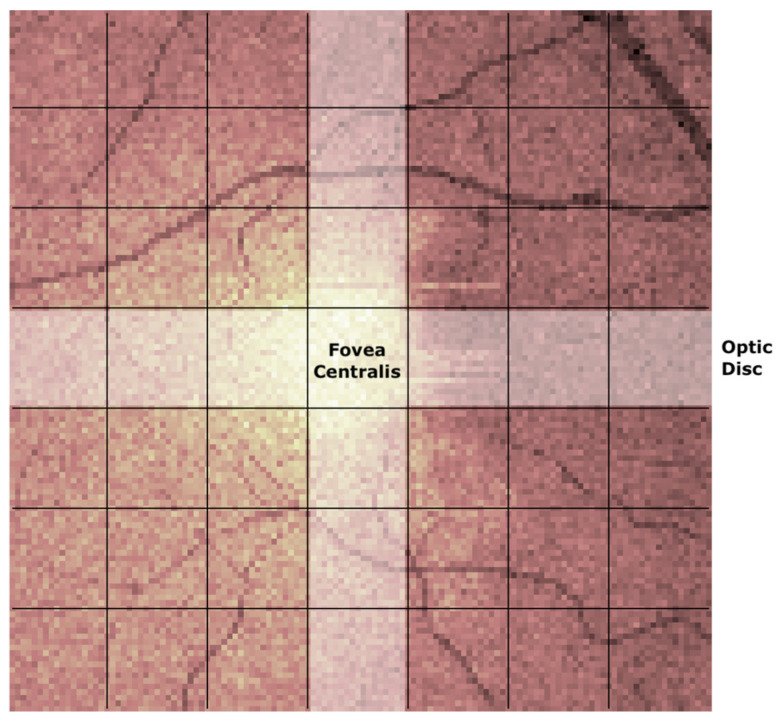
Computed mean value fundus image of the outer nuclear layer of the right eye of a 54-year old female participant. Each of the 7 × 7 blocks show the individually analysed areas which results were later aggregated into larger regions (shaded areas). Image axes are: *x*-axis (horizontal)—temporal (**left**) to nasal (**right**) and *y*-axis (vertical)—superior (**top**) to inferior (**bottom**).

Similar to our prior works [33,39], the down-sampled images were split into 7 × 7 non-overlapping blocks (block size: 18 pixels in both directions), which were independently analyzed. In each image, the central (4th) row and column of the 49-block grid square were discarded from the analysis to exclude the image region corresponding to the fovea, where some of the six retinal layers examined are reduced or inexistent (see Figure 4).

For each of the remaining 36 blocks on each image, four GLCMs were computed for the pixel-pair orientations of 0°, 45°, 90°, and 135°, with angles 180° apart considered the same, e.g., the pixel-pairs (i, j)/(i, j + d) and (i, j)/(i, j − d), corresponding to the angles 0° and 180°, respectively, are considered to contribute to the same angle (0°). All matrices were computed for a pixel distance of one (d = 1). 

For each block and pixel-pair orientation, 21 features were computed: (1) Autocorrelation, (2) Cluster Prominence, (3) Cluster Shade, (4) Contrast, (5) Correlation, (6) Difference Entropy, (7) Difference Variance, (8) Dissimilarity, (9) Energy, (10) Entropy, (11) Homogeneity, (12) Information Measure of Correlation 1 (IMC1), (13) Information Measure of Correlation 2 (IMC2), (14) Inverse Difference, (15) Inverse Difference Moment Normalised (IDMN), (16) Inverse Difference Normalized (IDN), (17) Maximum Probability, (18) Sum Average, (19) Sum Entropy, (20) Sum of Squares–Variance (SSV), and (21) Sum Variance. The definition of the texture features (1) to (3), (8), (10), and (17) can be found in [45], feature (14) in [46], and the remaining features in [36]—except features (15) and (16), which are simply normalized versions of features (11) and (14), respectively.

The maximum value across the four orientations for each feature in a given block was selected as the sole feature value for the block. Blocks were then aggregated into four groups of 3 × 3, corresponding to the temporal-superior (Q1), nasal-superior (Q2), temporal-inferior (Q3), and nasal-inferior (Q4) quadrants. Finally, the average value of each feature per retinal quadrant was used as the feature value for the quadrant. This resulted in a total of 504 texture features (21 GLCM features × 6 layers × 4 quadrants) being computed for each eye. 

### 2.4. Statistical Analysis

The computed texture features were tested for group mean differences between female and male participants’ right (OD) and left (OS) eyes. A pairwise correlation analysis was first performed to exclude similar features. 

Each feature was correlated (Pearson correlation) with all other features of the same eye and group (female OD and female OS; male OD and male OS). First, all feature pairs with a correlation coefficient |r|≥0.5 in both the right ($r_{OD}$) and the left ($r_{OS}$) eyes were identified separately for the female and male groups. Given the list of correlated features, they were then ordered by the decreasing number (n) of correlations with other features for which |r|≥0.5. In the case of a draw with respect to n, the largest sum of explained variance, given by $\sum_{i=1}^{n}r_{OD,i}^{2}+r_{OS,\;i}^{2}$, was considered. Features were selected following this sorted list, while all their correlated features (|r|≥0.5) were discarded. This process was repeated until two subsets of texture features (one for the female and the other for the male group) were obtained, where all pairwise correlation coefficients satisfied −0.5<r<0.5. 

Each non-correlated feature was first tested for normality within the same eye and group. The Shapiro–Wilk test was used at a 10% significance level for a more conservative normality decision. If the feature presented a normal distribution for both the right- and the left-eye groups, a paired-sample *t*-test was used to assess the group mean differences; otherwise, a Wilcoxon signed rank test was applied. 

Due to the large number of independent tests performed, we considered three corrections for multiple comparisons: Bonferroni, Benjamini–Hochberg, and the False Discovery Rate (FDR), as proposed by Storey [47]. 

## 3. Results

### 3.1. Pairwise Correlations

Out of 504 texture features, 146 (29.0%) and 148 (29.4%) were found to be non-correlated for the female and male groups, respectively. These features are listed in an additional file (see Supplementary Table S1).

### 3.2. Normality Testing

For female participants, a non-parametric test was applied to 73 out of 146 non-correlated texture features (50.0%), while for male participants, it was applied to 74 out of 148 features (50.0%).

### 3.3. Hypothesis Testing

**Figure 5:**
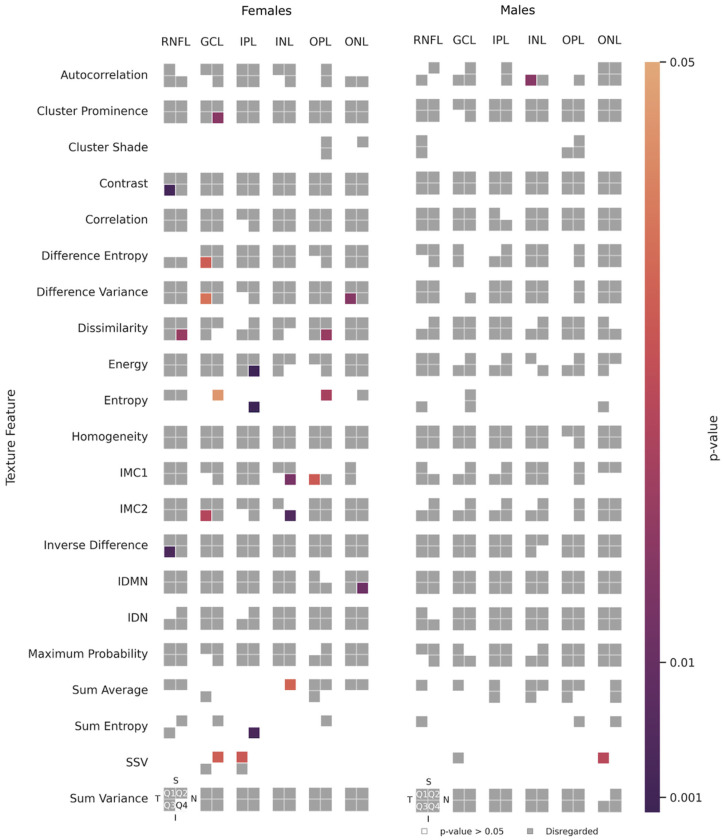
Texture features under study, for the female (**left**) and male (**right**) groups, for the retinal nerve fibre layer (RNFL), the ganglion cell layer (GCL), the inner plexiform layer (IPL), the inner nuclear layer (INL), the outer plexiform layer (OPL), and the outer nuclear layer (ONL). Q1 stands for the superior-temporal quadrant, Q2 stands for the superior-nasal quadrant, Q3 stands for the inferior-temporal quadrant, and Q4 stands for the inferior-nasal quadrant. In the features IMC1 and IMC2, IMC stands for Information Measure of Correlation; IDMN stands for Inverse Difference Moment Normalised; IDN stands for Inverse Difference Normalised, and SSV stands for Sum of Squares–Variance. The grey-coloured cells indicate features that can be disregarded (for they are correlated to other features). The colour-filled cells indicate non-correlated texture features with a *p*-value < 0.05 (significant difference). In contrast, the white/empty cells represent the non-correlated features with a *p*-value ≥ 0.05 (non-significant difference).

Of the 146 non-correlated texture features in the female group, 21 (14.4%) present statistical differences (*p* < 0.05 for each test) between the right and left eyes (left side of Figure 5). Seven (33.3%) of these 21 features are distributed in Q3, and 9 (42.9%) in Q4. Layer-wise, the GCL and IPL gather the highest number of differences: 6 (28.6%) and 4 (19.1%) features, respectively. Of the 21 features, two stand out: Energy (IPL/Q4) and Entropy (IPL/Q4), as they present results with a *p*-value < 0.001. In addition, six distinct features show a spread-out effect across different layers and quadrants of the retina: Dissimilarity (RNFL/Q4 and OPL/Q4), SSV(GCL/Q2 and IPL/Q1), Entropy (GCL/Q2, IPL/Q4, and OPL/ Q2), IMC2 (GCL/Q3 and INL/Q4), Difference Variance (GCL/Q3 and ONL/Q3), and IMC1 (INL/Q4 and OPL/Q3). 

Only two (1.4%) out of the 148 non-correlated texture features in the male group show statistical differences (right side of Figure 5): Autocorrelation (INL/Q3) and SSV (ONL/Q1). 

### 3.4. Multiple Comparison Corrections

When correcting the pairwise tests using the Bonferroni and the Benjamini–Hochberg correction, two statistically significant results are found in the female group: the Energy (IPL/Q4) and Entropy (IPL/Q4). No significant differences were found for the male group. 

**Figure 6:**
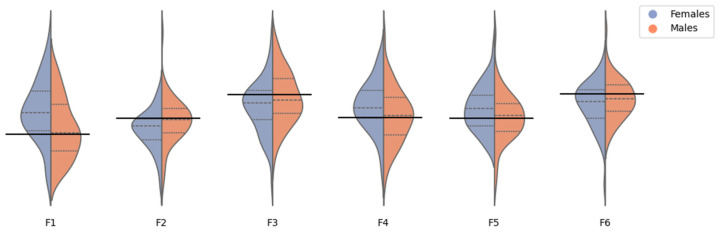
Kernel density estimation of the inter-eye differences for six features presenting differences between the left and right eye for women, but not for men. These features yield the most significant texture differences in the female group, even after Storey’s multiple comparisons correction. Features are: F1—Entropy (IPL/Q4); F2—Energy (IPL/Q4); F3—Contrast (RNFL/Q3); F4—Sum Entropy (IPL/Q4); F5—Inverse Difference (RNFL/Q3); and F6—IMC2 (INL/Q4). Features are ordered by increasing *p*-value, as per the female group analysis. The thick black line marks the zero.

Using Storey’s [47] method, the FDR was estimated to be ${\hat{FDR}}_{F}=13.8\%$ (female group) and ${\hat{FDR}}_{M}=100$% (male group). By calculating the *q*-values [47] and considering a 5% cut-off, six statistically significant differences (see Figure 6 for sample distributions) can be found in the female group (out of the 21 non-correlated features, i.e., 28.6%): (1) Contrast (RNFL/Q3), (2) Inverse Difference (RNFL/Q3), (3) Energy (IPL/Q4), (4) Entropy (IPL/Q4), (5) Sum Entropy (IPL/Q4), and (6) IMC2 (INL/Q4). No significant differences were found for the male group.

The number of statistically significant texture features in the female and male groups, before and after the three multiple comparisons correction methods were applied, are summarised in Table 2. 

Initial uncorrected results showed that interocular retinal texture asymmetries were mainly present in women and showed a larger effect size than in men. After correcting for multiple comparisons, all three correction methods revealed that interocular texture differences were, in fact, only present in the female group, while the eyes of men were similar.

## 4. Discussion

The present work focuses on sex differences between the right and left eye in healthy adults, as identified by neuroretina tissue texture analysis. The decision to specifically address sex differences was informed by our previous work [39], in which texture features revealed statistically significant differences between the retinas of the two sexes. Furthermore, texture features computed from the retina have been shown to be able to inform about the CNS status in different neurodegenerative disorders [24,25,33].

The rationale behind the analysis of these texture features is that each conveys quantitative information on the arrangement of the retina. Although a single texture feature, on its own, does not translate into a meaningful interpretation per participant/eye scan, we can quantify statistical differences between groups by combining several of these features. 

The hypothesis tests performed illustrate a clear pattern of significant differences between the two eyes in the female group, with texture differences spreading across all six layers of the neuroretina. In contrast, only a few significant differences were found in the male group. 

However, one must correct the pairwise tests due to the high number of comparisons. Considering the Benjamini–Hochberg correction, one finds the higher *k*, such that ${\tilde{p}}_{k}<\frac{k\alpha }{m\;}$, where ${\tilde{p}}_{k}$ are the ascending sorted *p*-values $p_{k}.$ Both this correction and the highly conservative Bonferroni method resulted in no statistically significant differences in the male group and two in the female group. 

We also considered the correction by FDR introduced by Storey [47], which is regarded as the most robust method of the three herein. In this approach, one starts by estimating the FDR in each group, resulting in the estimates ${\hat{FDR}}_{F}=13.8\%$ and ${\hat{FDR}}_{M}=100\%$. Although both estimates are above the desired 5%, it is clear that in the female group, the ${\hat{FDR}}_{F}$ is closer to the desired 5% than the ${\hat{FDR}}_{M}$, which is much larger. This indicates that some of the 21 significant differences (*p* < 0.05) found in the female group are indeed statistically significant.

In contrast, all the significant differences found in the male group are false positives. This conjecture is confirmed by calculating the *q*-values in both cases and considering a 5% cut-off, which results in six significant differences in the female group (from the 21 pairwise ones) and none in the male group. This way, the corrected results confirm that texture differences are found only in the female group. 

To the authors’ knowledge, the literature regarding the differences between the right and left eyes is mainly based on thickness assessments, and sex differences are seldom investigated in the healthy adult population—apart from [12], where a slight sex- and age-effect on retinal differences between the right and left eyes was reported. 

The strong point of this study is the reporting of a previously undocumented difference between women and men, which offers a unique contribution to determining normative sex differences. Texture metrics appear to be sensitive to these differences while raising the question of why such differences occur, how they might vary over the lifespan, and any potential evolutive advantage. A limitation of the present study is the absence of a stratified analysis per age group. However, such an analysis could not be performed because a reduced sample size in the study would negatively impact the statistical robustness of the reported results. 

This work highlights the potential of texture analysis to spot differences in the retina. Our findings suggest that special care should be taken when designing research protocols that assess retina differences, particularly when texture-based assessments are involved. On the one hand, the participant groups being compared should be sex-balanced—a good practice that was shown to be relevant in vision research [28]. On the other hand, beyond accounting for sex differences, studies should also control the interocular discrepancies that may be present in healthy individuals and patients diagnosed with ophthalmological or neurodegenerative disorders.

## Figures and Tables

**Table 1:** Demographic data.

	Female	Male
N	49	49
Age (years): mean(std)	42.5(16.3)	42.0(16.0)
Age (years): min(max)	19(74)	20(74)
Right (left) eyes	49(49)	49(49)
Total acquisitions	98	98

**Table 2:** Number of statistically significant texture features before and after correcting for multiple comparisons.

	Females	Males
Non-corrected results	21	2
Bonferroni	2	0
Benjamini-Hochberg	2	0
False Discovery Rate	6	0

## Data Availability

The data presented in this study are available on request from the corresponding author. The data are not publicly available because they were originally collected in the scope of different research projects.

## Supplementary Materials

The following supporting information can be downloaded at: https://www.mdpi.com/article/10.3390/jimaging10010006/s1, Table S1: Non-correlated texture features in the female and male groups, sorted by increasing *p*-value.

## References

[1] London A., Benhar I., Schwartz M. (2013). The retina as a window to the brain—From eye research to CNS disorders. Nat. Rev. Neurol..

[2] Svetozarskiy S., Kopishinskaya S. (2015). Retinal optical coherence tomography in neurodegenerative diseases (review). Sovrem. Tehnol. Med..

[3] Alves C., Jorge L., Canário N., Santiago B., Santana I., Castelhano J., Ambrósio A.F., Bernardes R., Castelo-Branco M. (2019). Interplay between macular retinal changes and white matter integrity in early Alzheimer’s disease. J. Alzheimers Dis..

[4] Czakó C., Kovács T., Ungvari Z., Csiszar A., Yabluchanskiy A., Conley S., Csipo T., Lipecz A., Horváth H., Sándor G.L. (2020). Retinal biomarkers for Alzheimer’s disease and vascular cognitive impairment and dementia (VCID): Implication for early diagnosis and prognosis. GeroScience.

[5] Vanston J.E., Strother L. (2017). Sex differences in the human visual system. J. Neurosci. Res..

[6] Hougaard A., Jensen B.H., Amin F.M., Rostrup E., Hoffmann M.B., Ashina M. (2015). Cerebral asymmetry of fMRI-BOLD responses to visual stimulation. PLoS ONE.

[7] Liu S., Zhao B., Shi C., Ma X., Sabel B.A., Chen X., Tao L. (2021). Ocular dominance and functional asymmetry in visual attention networks. Investig. Ophthalmol. Vis. Sci..

[8] El-Ashry M., Hegde V., James P., Pagliarini S. (2008). Analysis of macular thickness in British population using optical coherence tomography (OCT): An emphasis on interocular symmetry. Curr. Eye Res..

[9] Altan C., Arman B.H., Arici M., Urdem U., Solmaz B., Pasaoglu I., Basarir B., Onmez F., Taskapili M. (2019). Normative posterior pole asymmetry analysis data in healthy Caucasian population. Eur. J. Ophthalmol..

[10] Çubuk M., Kasım B., Koçluk Y., Sukgen E.A. (2018). Effects of age and gender on macular thickness in healthy subjects using spectral optical coherence tomography/scanning laser ophthalmoscopy. Int. Ophthalmol..

[11] Ly A., Banh J., Luu P., Huang J., Yapp M., Zangerl B. (2019). Interocular asymmetry of the superonasal retinal nerve fibre layer thickness and blood vessel diameter in healthy subjects. PLoS ONE.

[12] Jacobsen A.G., Bendtsen M.D., Vorum H., Bøgsted M., Hargitai J. (2015). Normal value ranges for central retinal thickness asymmetry in healthy Caucasian adults measured by SPECTRALIS SD-OCT posterior pole asymmetry analysis. Investig. Ophthalmol. Vis. Sci..

[13] Al-Haddad C., Antonios R., Tamim H., Noureddin B. (2014). Interocular symmetry in retinal and optic nerve parameters in children as measured by spectral domain optical coherence tomography. Br. J. Ophthalmol..

[14] Pawar N., Maheshwari D., Ravindran M., Ramakrishnan R. (2017). Interocular symmetry of retinal nerve fiber layer and optic nerve head parameters measured by Cirrus high-definition optical coherence tomography in a normal pediatric population. Indian J. Ophthalmol..

[15] Song M.Y., Hwang Y.H. (2022). Interocular symmetry of optical coherence tomography parameters in healthy children and adolescents. Sci. Rep..

[16] Adhi M., Aziz S., Muhammad K., Adhi M.I. (2012). Macular thickness by age and gender in healthy eyes using spectral domain optical coherence tomography. PLoS ONE.

[17] Ooto S., Hangai M., Tomidokoro A., Saito H., Araie M., Otani T., Kishi S., Matsushita K., Maeda N., Shirakashi M. (2011). Effects of age, sex, and axial length on the three-dimensional profile of normal macular layer structures. Investig. Ophthalmol. Vis. Sci..

[18] Ooto S., Hangai M., Yoshimura N. (2015). Effects of sex and age on the normal retinal and choroidal structures on optical coherence tomography. Curr. Eye Res..

[19] Delori F.C., Goger D.G., Keilhauer C., Salvetti P., Staurenghi G. (2006). Bimodal spatial distribution of macular pigment: Evidence of a gender relationship. J. Opt. Soc. Am. A.

[20] Haan J., Verbraak F.D., Visser P.J., Bouwman F.H. (2017). Retinal thickness in Alzheimer’s disease: A systematic review and meta-analysis. Alzheimers Dement. Diagn. Assess. Dis. Monit..

[21] Hart N.J., Koronyo Y., Black K.L., Koronyo-Hamaoui M. (2016). Ocular indicators of Alzheimer’s: Exploring disease in the retina. Acta Neuropathol..

[22] Archibald N.K., Clarke M.P., Mosimann U.P., Burn D.J. (2009). The retina in Parkinson’s disease. Brain.

[23] Tian T., Zhu X.H., Liu Y.H. (2011). Potential role of retina as a progression of Parkinson’s disease. Int. J. Ophthalmol..

[24] Varga B.E., Gao W., Laurik K.L., Tátrai E., Simó M., Somfai G.M., DeBuc D.C. (2015). Investigating tissue optical properties and texture descriptors of the retina in patients with multiple sclerosis. PLoS ONE.

[25] Tazarjani H.D., Amini Z., Kafieh R., Ashtari F., Sadeghi E. (2021). Retinal OCT texture analysis for differentiating healthy controls from multiple sclerosis (MS) with/without optic neuritis. BioMed Res. Int..

[26] Nuzzi R., Scalabrin S., Becco A., Panzica G. (2018). Gonadal hormones and retinal disorders: A review. Front. Endocrinol..

[27] Nuzzi R., Caselgrandi P. (2022). Sex hormones and their effects on ocular disorders and pathophysiology: Current aspects and our experience. Int. J. Mol. Sci..

[28] Shaqiri A., Roinishvili M., Grzeczkowski L., Chkonia E., Pilz K., Mohr C., Brand A., Kunchulia M., Herzog M.H. (2018). Sex-related differences in vision are heterogeneous. Sci. Rep..

[29] Schuman J., Orgul S., Gugleta K., Dubler B., Flammer J. (2000). Interocular difference in progression of glaucoma correlates with interocular differences in retrobulbar circulation. Am. J. Ophthalmol..

[30] Xu G., Hu Y., Zhu S., Guo Y., Xiong L., Fang X., Liu J., Zhang Q., Huang N., Zhou J. (2021). A multicenter study of interocular symmetry of corneal biometrics in Chinese myopic patients. Sci. Rep..

[31] Cava J.A., Allphin M.T., Mastey R.R., Gaffney M., Linderman R.E., Cooper R.F., Carroll J. (2020). Assessing interocular symmetry of the foveal cone mosaic. Investig. Ophthalmol. Vis. Sci..

[32] Anantrasirichai N., Achim A., Morgan J.E., Erchova I., Nicholson L. SVM-based texture classification in optical coherence tomography. Proceedings of the 2013 IEEE 10th International Symposium on Biomedical Imaging.

[33] Nunes A., Silva G., Duque C., Januário C., Santana I., Ambrósio A.F., Castelo-Branco M., Bernardes R. (2019). Retinal texture biomarkers may help to discriminate between Alzheimer’s, Parkinson’s, and healthy controls. PLoS ONE.

[34] Julesz B. (1962). Visual pattern discrimination. IRE Trans. Inf. Theory.

[35] Bergen J.R., Landy M.S., Landy M., Movshon J.A. (1991). Computational modeling of visual texture segregation. Computational Models of Visual Processing.

[36] Haralick R.M., Shanmugam K., Dinstein I.H. (1973). Textural features for image classification. IEEE Trans. Syst. Man Cybern..

[37] Tourassi G.D. (1999). Journey toward computer-aided diagnosis: Role of image texture analysis. Radiology.

[38] Castellano G.L., Bonilha L., Li L.M., Cendes F. (2004). Texture analysis of medical images. Clin. Radiol..

[39] Nunes A., Serranho P., Quental H., Ambrósio A.F., Castelo-Branco M., Bernardes R. (2020). Sexual dimorphism of the adult human retina assessed by optical coherence tomography. Health Technol..

[40] World Medical Association (2013). World Medical Association Declaration of Helsinki: Ethical principles for medical research involving human subjects. JAMA.

[41] Li K., Wu X., Chen D., Sonka M. (2005). Optimal surface segmentation in volumetric images—A graph-theoretic approach. IEEE Trans. Pattern Anal. Mach. Intell..

[42] Garvin M.K., Abramoff M.D., Wu X., Russell S.R., Burns T.L., Sonka M. (2009). Automated 3-D intraretinal layer segmentation of macular spectral-domain optical coherence tomography images. IEEE Trans. Med. Imaging.

[43] Abràmoff M.D., Garvin M.K., Sonka M. (2010). Retinal imaging and image analysis. IEEE Rev. Biomed. Eng..

[44] Guimarães P., Rodrigues P., Lobo C., Leal S., Figueira J., Serranho P., Bernardes R. (2014). Ocular fundus reference images from optical coherence tomography. Comput. Med. Imaging Graph..

[45] Soh L.-K., Tsatsoulis C. (1999). Texture analysis of SAR sea ice imagery using gray level co-occurrence matrices. IEEE Trans. Geosci. Remote Sens..

[46] Clausi D.A. (2002). An analysis of co-occurrence texture statistics as a function of grey level quantization. Can. J. Remote Sens..

[47] Storey J.D. (2002). A direct approach to false discovery rates. J. R. Stat. Soc. Ser. B.

